# Acute Heat Stress and Reduced Nutrient Intake Alter Intestinal Proteomic Profile and Gene Expression in Pigs

**DOI:** 10.1371/journal.pone.0143099

**Published:** 2015-11-17

**Authors:** Sarah C. Pearce, Steven M. Lonergan, Elisabeth Huff-Lonergan, Lance H. Baumgard, Nicholas K. Gabler

**Affiliations:** Department of Animal Science, Iowa State University, Ames, Iowa, United States of America; University of Lleida, SPAIN

## Abstract

Heat stress and reduced feed intake negatively affect intestinal integrity and barrier function. Our objective was to compare ileum protein profiles of pigs subjected to 12 hours of HS, thermal neutral ad libitum feed intake, or pair-fed to heat stress feed intake under thermal neutral conditions (pair-fed thermal neutral). 2D-Differential In Gel Electrophoresis and gene expression were performed. Relative abundance of 281 and 138 spots differed due to heat stress, compared to thermal neutral and pair-fed thermal neutral pigs, respectively. However, only 20 proteins were different due to feed intake (thermal neutral versus pair-fed thermal neutral). Heat stress increased mRNA expression of heat shock proteins and protein abundance of heat shock proteins 27, 70, 90-α and β were also increased. Heat stress reduced ileum abundance of several metabolic enzymes, many of which are involved in the glycolytic or TCA pathways, indicating a change in metabolic priorities. Stress response enzymes peroxiredoxin-1 and peptidyl-prolyl cis-trans isomerase A were decreased in pair-fed thermal neutral and thermal neutral pigs compared to heat stress. Heat stress increased mRNA abundance markers of ileum hypoxia. Altogether, these data show that heat stress directly alters intestinal protein and mRNA profiles largely independent of reduced feed intake. These changes may be related to the reduced intestinal integrity associated with heat stress.

## Introduction

Heat stress exposure markedly increases respiration rates and body temperatures, slows body weight gains and significantly reduces performance in humans and livestock [1–4]. To global animal production alone, the effects of high ambient temperatures have been estimated to cause losses to animal agriculture that are in the billions of dollars annually [5]. Interestingly, besides cooling and rehydration, there are few standard medical procedures to treat heat stroke and for patients admitted to hospitals mortality is thought to be >30% [6]. Of the various organs in the body, the gastrointestinal tract is highly sensitive to heat stress and a compromised intestinal mucosa plays a critical role in the pathophysiology of hyperthermia as observed in multiple models [7,8].

The gastrointestinal tract primarily serves the important functions of selectively absorbing nutrients and water from the lumen and forming a critical barrier between the luminal contents and systemic circulation. Alterations in this function and integrity will be detrimental to animal health, performance and wellbeing [9]. Heat stress and heat stroke can cause intestinal dysfunction and increases gastrointestinal permeability to macromolecules and endotoxin [10–12]. Moreover, heat stress has been shown to increase pathogenic Salmonella migration across the intestinal tract of poultry [13].

Physiologically, the intestines are impacted during heat-stress due to the partitioning of blood to the periphery in an attempt to maximize radiant heat dissipation. This blood redistribution is supported by vasoconstriction of the gastrointestinal tract [14,15]. As a result, reduced blood and nutrient flow causes hypoxia at the intestinal epithelium, which ultimately compromises intestinal integrity and function [16]. Intestinal function and integrity can be altered by inflammation and hypoxia, which are known to regulate intestinal tight junction (TJ) proteins such as occludin and claudin [17–19]. However, although we have consistently observed an up-regulation of intestinal hypoxia markers in heat stressed pigs, we have not observed heat stress induced changes in intestinal inflammation [11,20]. Moreover, reduced nutrient and caloric intake during high ambient temperature exposure may also significantly contribute to this compromised intestinal integrity [7].

Stress and reduced enteral caloric intake has been shown to compromise intestinal mucosal integrity in pigs [21]. Mammals subjected to heat stress conditions often reduced feed consumption to minimize the thermal effect of digestion [22–25]. To study this, we have previously reported using a seven day swine pair-feeding model matched to heat stress pig feed intake levels. Many of the changes due to heat stress within the gastrointestinal tract appear to be directly mediated by reduced feed intake. Consequently, it is important to understand not just how higher core temperatures alter intestinal function, but also the role of reduced feed intake [12].

Altogether, given these observations, our objective was to evaluate the consequences of an acute, severe heat-load and reduced feed intake on the ileum protein profile using two-dimensional DIGE and MALDI-TOF mass spectrometry. We also aimed to identify intestinal protein profiles that may explain how pigs perceive and initially adapt to extreme ambient heat loads. Additionally, we aimed to examine gene abundance differences related to ileum function, metabolism and integrity of these pigs to further identify pathway changes resulting from heat stress.

## Materials and Methods

### Animals and study design

All experimental procedures involving animals used in this experiment have been previously described [20] and approved by the Iowa State University Institutional Animal Care and Use Committee (protocol #2-12-7307-S). Briefly, crossbred gilts (*n* = 24, 64 ± 3 kg body weight; Pig Improvement Company C22/C29 × 337) were selected by BW and randomly blocked into individual pens (with individual feeders and waters) across two rooms. Animals were allowed to acclimate to their pens for 3 d and were reared in thermal neutral (TN) conditions (20 ± 1°C; 35 to 50% relative humidity). Thereafter, pigs were then assigned to one of three treatments for 12 h: 1) thermal neutral (TN, n = 8) conditions (21°C; 70% humidity) with ad libitum feed intake; 2) heat stress (HS, n = 8) conditions (constant 37°C; 40% humidity) with ad libitum intake; or 3) pair-fed thermal neutral (PFTN, n = 8) conditions to mirror feed intake of the HS pigs while reared under TN conditions. Pair-feeding was conducted to quantify confounding effects of dissimilar nutrient intake as we have previously described [23,26]. During the 12 h period, animals were monitored continuously for signs of distress and measures such as core temperature and respiration rates were taken every 2 h.

### Tissue collection and protein extraction

After the 12 h experimental period, all pigs were euthanized by barbiturate overdose. A section of the ileum (2 m proximal from the ileal-cecal junction) was collected, flushed with phosphate-buffered saline (PBS) and snap frozen in liquid nitrogen, and stored at -80°C until further analysis.

Ileal samples (0.5 g) were homogenized in 4 mL of a urea buffer (8.3 M urea, 2 M thiourea, 2% CHAPS, and 1% DTT, pH 8.5), homogenized, and then centrifuged at 10,000 x g for 30 min. The supernatant was reserved and protein concentrations were determined using the Quick Start^™^ Bradford Protein Assay (BioRad, Hercules, CA). Samples were then adjusted to a final protein concentration of 3.5 mg/mL.

### Two-dimensional difference in gel electrophoresis

Two-dimensional difference in gel electrophoresis (2D-DIGE) was used as previously described [27] to determine differences in protein profile in whole ileum between TN, HS, and PFTN animals. A pooled reference was created from equal protein amounts from all animals, and then the reference was used for protein identification gels. A total of 50 μg of each individual sample was labeled with CyDyes 3 or 5 (GE Healthcare, Piscataway, NJ) according to the manufacturer's directions. As only two samples plus a reference could be run on one gel, the CyDyes 3 and 5 were alternated between the three treatments and arranged in such a way that all combinations (i.e. TN vs. HS, PFTN vs. HS and TN vs. PFTN) were covered. CyDye 2 was used to label the pooled standard sample. For each individual run, 15 μg of labeled ileal protein (CyDye 3 or 5) was used in addition to 15 μg of the pooled standard (CyDye 2) for a total of 45 μg of protein per gel.

For the first dimension, DeStreak Rehydration Solution (GE Healthcare, Piscataway, NJ) with 2.5 mM dithiothreitol (DTT) was added to the protein mixture to the volume specified by strip manufacturer (200 μL). The protein mixture was added to individual wells in a re-swelling tray. Immobilized pH gradient (IPG) strips (11 cm, pH 3–10, GE Healthcare, Piscataway, NJ) were placed on the protein mixture in the wells and allowed to rehydrate overnight at room temperature in a humidified chamber. Isoelectric focusing was performed using an Ettan IPGphor isoelectric focusing system (GE Healthcare, Piscataway, NJ) for a total of 11,500 V/h. After isoelectric focusing, strips were equilibrated using two 15 min washes; first with equilibration buffer (50 mM Tris–HCl pH 8.8, 6 M urea, 30% glycerol, 2% SDS, and trace amounts of bromophenol blue) containing 65 mM DTT; and second with equilibration buffer containing 135 mM iodoacetamide.

The equilibrated strips were loaded onto 12.5% SDS-PAGE gels (100:1 acrylamide:N,N′-bis-methylene acrylamide, 0.1% SDS, 0.05% TEMED, 0.05% ammonium persulfate, and 0.5 M Tris–HCL, pH 8.8) using agarose as an overlay to hold the strips in place. The ileum protein samples were then run in the second dimension using an Ettan DALT SIX system (GE Healthcare, Piscataway, NJ) containing 24 cm gels. Two 11 cm strips were placed side-by-side endwise on each gel. Each gel was run in duplicate for a total of 48 gels. Gels were imaged using an Ettan DIGE Imager (GE Healthcare, Piscataway, NJ). Images were processed and analyzed using DeCyder 2D software version 6.5 (GE Healthcare, Piscataway, NJ) as previously described [27].

### Protein identification

Spots were identified as being differentially abundant using fold change, which was then calculated to percentage change, and two way ANOVA analysis (*P* < 0.05) by the DeCyder 2D software between treatments (TN vs HS; HS vs PFTN; and TN vs PFTN) were selected for identification. To identify these proteins, unlabeled pooled ileal protein references (500–750 μg) were resolved using 2DE and stained with Colloidal Coomassie Blue Stain (1.7% ammonium sulfate, 30% methanol, 3% phosphoric acid, and 0.1% Coomassie G-250) for 48 h prior to destaining in water for 24 h. Spots identified as different in the DeCyder analysis that were not prominent on the Colloidal Coomassie stained gel were not selected for identification. To reduce potential for contamination all reagents and buffers used during the protein identification step were filtered (0.22 μm) prior to use.

Selected spots were excised from the 2D gel and sent to the Iowa State University Protein Facility for identification. In-gel trypsin digest using Genomics Solution ProGest (Chelmsford, MA) was performed and the peptides dissolved in CHCA (5 mg/ml in 50% CH3CN/0.1% TFA) before being deposited to a MALDI target. MALDI Mass Spectrometry was performed using a QSTAR XL Quad- rupole TOF mass spectrometer equipped with an oMALDI ion source (AB/MDS Sciex, Toronto, Canada). A peak list was generated by Analyst QS Version 1.1 (AB/MDS Sciex, Toronto, Canada). Spectra were processed by MASCOT database search Version 2.2.07 (MatrixScience, London, UK). Search conditions included maximum one missed cleavage, fixed modification (carboxyamidomethyl cysteine), variable modification (oxidation of methionine), peptide mass tolerance of ± 100 ppm, and fragment mass tolerances of ±1 Da. Identification was based on Mowse Score with a threshold of less than 0.05. Confirmation of five proteins was carried out using 2D western blotting technique described previously [28] using a 7 cm pH 3–10 strip, and a 12.5% second dimension gel. The antibodies used for this conformation are listed in S1 Table.

### mRNA abundance analysis

Total RNA from all tissue samples used for protein isolation was isolated from 0.2 g of whole frozen ileum samples using a commercially available kit and its protocol (RNeasy fibrous tissue mini kit, Qiagen, Valencia, CA). Total RNA was quantified and cDNA synthesized for real-time quantitative PCR as previously described [12]. Real-time quantitative PCR was performed on one 48.48 Dynamic Array IFC plate to analyze mRNA abundance using a BioMark^™^ HD system (Fluidigm Corporation, San Francisco, CA). The ileal derived cDNA used underwent specific target amplification using the TaqMan PreAmp Master Mix (Life Technologies) and was loaded onto Fluidigm’s Dynamic Array Integrated Fluidic Circuits (IFC) according to Fluidigm’s EvaGreen DNA binding Dye protocols (Fluidigm Corporation, San Francisco, CA). Gene symbols, accession numbers and primer sequences are listed in S2 Table. TOP2B was included into the quantitative PCR array as an endogenous reference gene and used for calculation. The mRNA abundance values for each sample were normalized to this reference genes using the 2^ΔΔCT^ method [29] and the Fluidigm Biomark^™^ HD software (Fluidigm Corporation, San Francisco, CA).

### Statistical analysis

Protein spot differences were statistically analyzed by two-way ANOVA using the DeCyder 2D software version 6.5 (GE Healthcare, Piscataway, NJ) and reported as percentage change from either thermal neutral or pair-fed thermal neutral treatments. Gene abundance data were statistically analyzed using the PROC MIXED procedure of SAS version 9.2 (SAS Inst. Inc. Cary, NC). Data are reported as LSmeans and considered significant if *P* ≤ 0.05 and a tendency if 0.05 > *P* ≤ 0.10. The model included comparison of all 3 treatments (TN, HS and PFTN) as a fixed effect.

## Results

### Pig phenotypic response to heat stress

The phenotypic responses to heat stress and pair-feeding are described in Table 1 and the functionality data has been reported in Pearce et al., [30]. As expected, HS increased rectal temperature and respiration rates compared to both the TN and PFTN pigs (*P* < 0.01). Further, HS reduced the 12 h ad libitum feed intake by 90% in HS compared to the TN control pigs (*P* < 0.01). This reduction in feed intake contributed to the significant (*P* < 0.01) 12 h loss of body weight in the HS and PFTN pigs.

**Table 1:** Effects of 12 h of thermal neutral (TN; 21°C) ad libitum, pair-feeding in thermal neutral conditions (PFTN) or heat stress (HS; 37°C) ad libitum conditions on pig phenotypic measures.

Parameter	Treatment			SEM	*P-value*
	TN	PFTN	HS		
Rectal Temperature, °C	39.2^a^	39.1^a^	41.8^b^	0.10	<0.01
Respiration Rate, bpm	42.1^a^	41.9^a^	154.3^b^	2.70	<0.01
Feed Intake, kg	1.06^a^	0.18^b^	0.11^b^	0.082	<0.01
Δ Body weight, kg	0.39^a^	-1.87^b^	-4.56^c^	0.431	<0.01

^a,b,c^ *P* < 0.05,

n = 8/treatment

### Ileal protein profile changes in response to heat stress or pair-feeding

**Fig 1:**
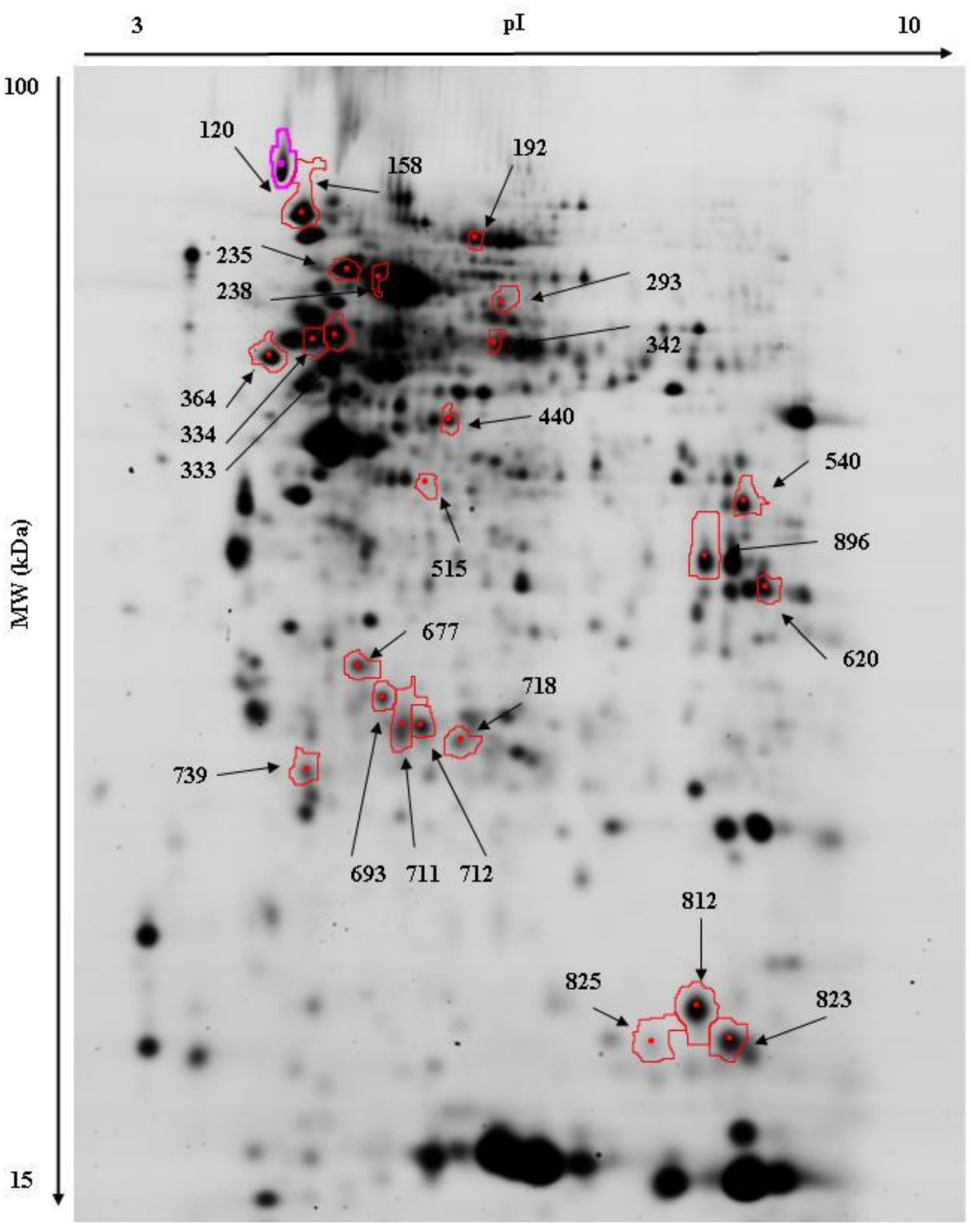
A representative 2D-DIGE gel from the ileum showing identified proteins. A total of 45 μg of CyDye labeled protein (15 μg of each CyDye 2, 3, and 5) was loaded onto a 11 cm pH 3–10 IPG strip for the first dimension and the second dimension was run on a 12.5% SDS-PAGE gel. Proteins labeled with CyDye5 are shown.

**Fig 2:**
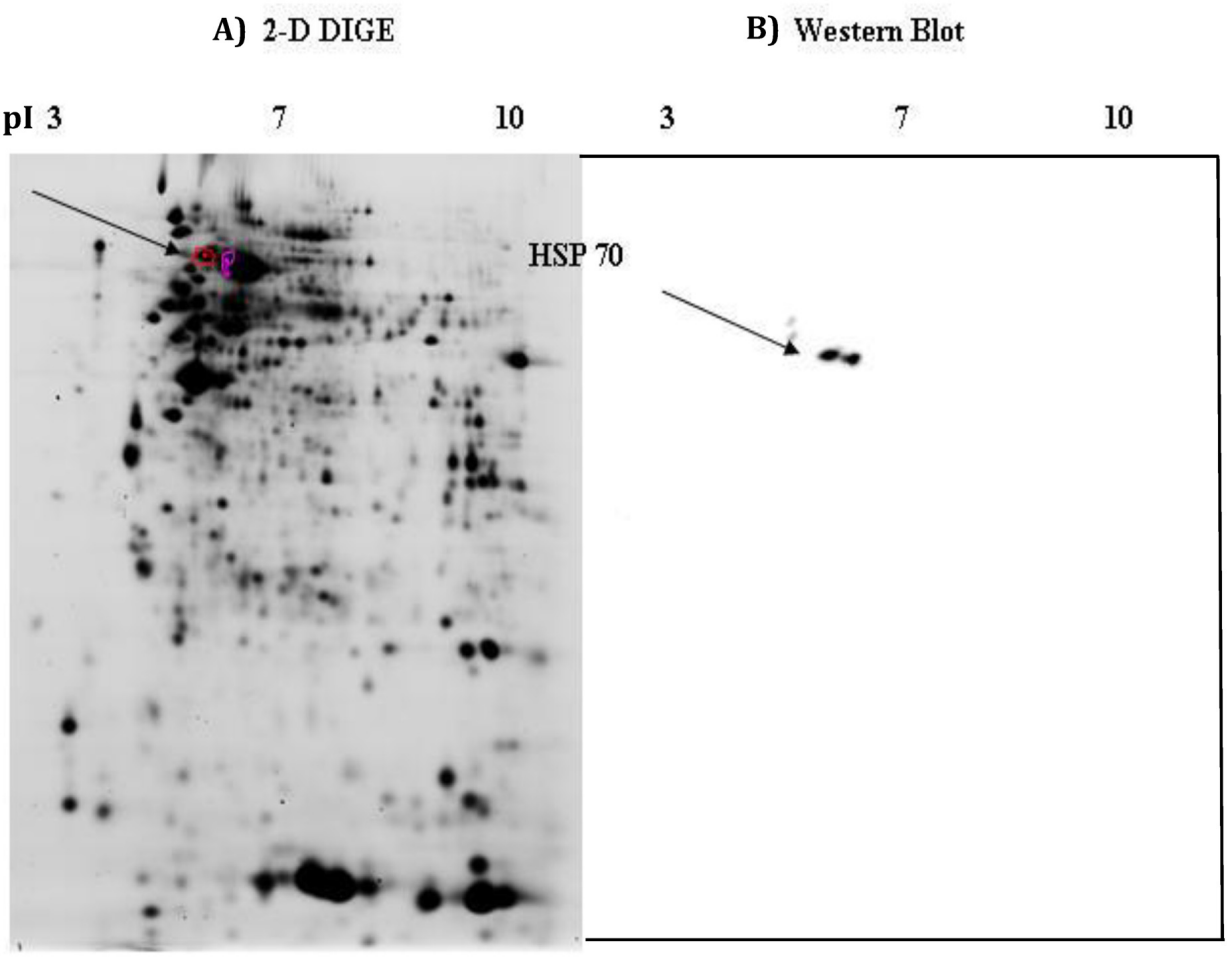
Detection of heat shock protein 70 (HSP 70) in ileum by the comparison of a (A) 2D-DIGE image (CyDye 5) and (B) western blot. 2D-DIGE performed using 15 μg of protein labeled with each CyDye loaded on an 11 cm pH 3–10 strip for the first dimension and the second dimension on a 12.5% SDS-PAGE gel (acrylamide: N,N′-bis-methylene acrylamide 100:1, 0.1% SDS, 0.05% TEMED, 0.05% ammonium persulfate, and 0.5 M Tris–HCL, pH 8.8). Western blot protocols were followed after loading 40 μg of protein on a 7 cm 3–10 pH strip and running the second dimension on a 12.5% SDS-PAGE gel.

A total of 899 protein spots were discovered in the isolated ileum tissue by 2D-DIGE. These spots were then filtered based upon appearance (detected on >50% of gels) and the final spot count was adjusted to 551. Twenty spots were identified as differentially abundant (*P* < 0.05) between TN and PFTN pigs (8 increased due to PFTN). Two hundred eighty one spots were differentially abundant (*P* < 0.05) between TN and HS pigs (111 increased due to HS). One hundred thirty eight spots were differentially abundant (*P* < 0.05) between PFTN and HS pigs (45 increased due to HS). Based upon *P*-value rankings, we selected a total of 30 protein spots that were picked for identification, of which 24 were identified (Fig 1; Tables 2 and 3). Confirmation of five of these 24 proteins (aldolase, HSP 27, HSP 70, GAPDH, and HSP 90-α) was carried out using a 2D western blotting technique (Fig 2; S1 Fig, S1 Table).

**Table 2:** Differentially abundant proteins between thermal neutral (TN) and heat-stressed (HS) pigs at 12 h.

Spot#	Protein^a^	Swiss-Prot name	Sequence Coverage	Pi (theo/exp) heo/exp)	MW (kDa) (theo/exp)	**% Change** ^b^	***P*-value**
515	Isocitrate Dehydrogenase [NADP], mitochondrial	IDH1_PIG	12%	8.74/9.20	48/47	-100	<0.0001
158	Heat shock protein 90-α	HS90A_PIG	7%	4.93/4.94	85/85	40	<0.0001
120	HSP 90-β-1	HS90B_MOUSE	9%	4.74/4.97	93/83	20	0.0007
364	Prolyl 4-hydroxylase-β	P4HB _PIG	9%	4.78/4.82	57/57	-10	0.0001
334	Vimentin	VIME_PIG	8%	5.06/5.06	54/54	20	0.002
718	Heat shock protein β-1	HSPB1_PIG	24%	6.23/5.98	23/23	20	0.0015
823	Peptidyl-prolyl cis-trans isomerase A	PPIA_PIG	14%	8.33/7.68	18/18	-20	<0.0001
825	Peptidyl-prolyl cis-trans isomerase A	PPIA_PIG	19%	8.34/	18/18	-16	<0.0001
711	Peroxiredoxin-1	PRDX1_PIG		/8.27	/22	-50	<0.0001
739	Rho GDP inhibitor 1	GDIR_BOVIN	11%	5.12/5.03	24/23	-15	<0.0001
192	Neurofibromin-1	NF1_RAT	10%	6.94/7.10	32/220	14	<0.0001
235	Heat shock protein 70	HSP70_PIG	8%	5.37/5.48	71/70	18	<0.0001
238	Heat shock protein 70	HS71A_MOUSE	10%	5.92/5.53	71/70	92	<0.0001
333	Heat shock protein 65	CH60_MOUSE	6%	5.91/	61/57	9	0.0047
440	Alpha-enolase	ENO1_PIG	15%	6.37/7.01	48/47	-12	<0.0001
540	Fructose Bisphosphate Aldolase	ALDOA_RABIT	12%	8.30/8.47	40/40	-11	<0.0001
293	Stress-induced phosphoprotein 1	STIP1_BOVIN	3%	6.08/6.40	63/63	29	0.0002
812	Cofilin-1	COF1_PIG	13%	8.16/8.22	19/19	-16	<0.0001
620	Calponin-1	CNN1_PIG	10%	8.91/8.92	33/34	-13	0.0420
693	Proteasome activator PA28-α subunit	PSME1 _PIG	14%	5.45/5.78	24/28	-12	0.0015
677	Proteasome activator complex subunit 2	PSME2_PIG	9%	5.41/5.54	27/28	-9	0.0043
342	Immunoglobulini G γ-chain	IGHG_PIG	6%	6.71/6.40	52/55	11	0.0004
896	Glyceraldehyde-3-phosphate dehydrogenase (phosphorylating)	G3P_PIG	12%	6.90/8.57	36/36	-4	0.0018

^a^ Trypsin used to digest all proteins

^b^ Positive values indicate an increase in HS pigs. Negative values indicate decrease in HS pigs.

**Table 3:** Differentially abundant proteins between pair-fed thermal neutral (PFTN) and heat-stressed (HS) pigs at 12 h.

Spot ID	Protein^a^	Swiss-Prot name	Sequence Coverage	Pi (theo/exp)	MW (kDa) (theo/exp)	% Change^b^	*P*-value
515	Isocitrate Dehydrogenase [NADP], mitochondrial	IDH1_PIG	12%	8.74/9.10	48/	-70	<0.0001
158	Heat shock protein 90-α	HS90A_PIG	7%	4.93/4.94	85/	40	<0.0001
718	Heat shock protein β-1	HSPB1_PIG	24%	6.23/5.98	23/	20	0.0015
823	Peptidyl-prolyl cis-trans isomerase A	PPIA_PIG	14%	8.33/7.68	18/	-10	<0.0001
711	Peroxiredoxin-1	PRDX1_				-30	<0.0001
293	Stress-induced-phosphoprotein 1	STIP1_BOVIN	3%	6.08/	63/	32	0.0002
120	Endoplasmin precursor (HSP 90-β)	HS90B_MOUSE	9%	4.74/4.82	93/	19	0.0007
235	Heat shock protein 70 (HSP 70)	HS71A_MOUSE	8%	5.37/5.48	71/	20	<0.0001
192	Neurofibromin 1 (NF-1)	NF1_RAT	10%	6.94/7.10	32/	20	<0.0001
342	Immunoglobulin γ-chain	IGHG_PIG	6%	6.71/6.40	52/	18	0.0004
333	Heat shock protein 65	CH60_MOUSE	6%	5.91/	61/	10	0.0047
896	Glyceraldehyde-3-phosphate dehydrogenase (phosphorylating)	G3P_PIG	12%	6.90/8.57	36/	-8	0.0018
540	Fructose 1,6-Bisphosphate Aldolase	ALDOA_RABIT	12%	8.30/8.47	40/	-8	<0.0001
440	Alpha-enolase	ENO1_PIG	15%	6.37/7.01	48/	-10	<0.0001
739	Rho GDP inhibitor 1 (GDIR1)	GDIR_BOVIN	11%	5.12/5.03	24/	-14	<0.0001
812	Cofilin-1	COF1_PIG	13%	8.16/8.22	19/	-14	<0.0001
825	Peptidyl-prolyl cis-trans isomerase A	PPIA_PIG	19%	8.34/7.68	18/	-15	<0.0001

^a^ Trypsin used to digest all proteins

^b^ Positive values indicate an increase in HS pigs. Negative values indicate decrease in HS pigs.

In the comparison of TN vs. HS (Table 2), several heat shock proteins (HSP’s) were upregulated in the HS treatments including HSP 27 (20% increase), HSP 90-α (40% increase), HSP 90-β (20% increase), HSP 70 (2 spots one increased 92% and the other 18%), HSP 65 (9% increase). Metabolic proteins that were differentially abundant at a *P* < 0.05 due to HS treatment compared to TN included mitochondrial isocitrate dehydrogenase (2-fold decrease), glyceraldehyde-3-phosphate dehydrogenase (GAPDH; 4% decrease), and fructose 1,6-bisphosphate aldolase (11% decrease, alpha-enolase (12% decrease). One protein related to oxidative stress that was differentially abundant includes peroxiredoxin-1 (50% decrease). Other stress and immune related proteins included prolyl 4-hydroxlase β (10% decrease), vimentin (20% increase), peptidyl-prolyl cis-trans isomerase (2 spots; 20 and 16% decreased), Rho GDP inhibitor 1 (50% decrease), neurofibromin (14% increase), stress-induced phosphoprotein 1 (29% increase), cofilin 1 (16% decrease), calponin 1 (13% decrease), proteasome activator PA28-α subunit (9% decrease), proteasome activator complex subunit 2 (9% decrease), and immunoglobulin G γ-chain (11% increase).

In the comparison of HS vs. PF (Table 3), many of the same proteins were identified as different as were in the TN vs. HS comparison. HSP 27 was increased 20% in HS pigs compared to PFTN counterparts. Another HSP, HSP 90-α, was also increased 40% in HS pigs, endoplasmin precursor (HSP 90-β) was increased 19%, HSP 65 was increased 10%, and HSP 70 was increased 20% in HS pigs. Metabolic proteins that were differentially abundant at a *P* < 0.05 in HS compared to PFTN included mitochondrial isocitrate dehydrogenase, (70% decrease), glyceraldehyde-3-phosphate dehydrogenase (GAPDH; 8% decrease), fructose 1,6-bisphosphate aldolase (8% decrease), and alpha enolase (10% decrease). The protein peroxiredoxin-1 which is related to oxidative stress that was decreased 30% in HS vs PFTN pigs. Compared to PFTN, HS also resulted in differential protein abundance of other stress and immune related proteins including peptidyl-prolyl-cis-trans isomerase A (2 spots at 10 and 15% decrease in HS), stress-induced phosphoprotein 1 (19% increase in HS), immunoglobulin γ-chain (18% increase in HS), Rho-GDP inhibitor 1 (14% decrease in HS), cofilin 1 (19% decrease in HS), and neurofibromin 1 (20% increase in HS).

In the comparison of TN vs. PF (data not shown), three proteins were identified as different. Prolyl-4-hydroxylase β polypeptide was decreased 10% in PFTN pigs compared to TN pigs, vimentin was increased 20%, and HSP 70 was increased 15% in PFTN pigs.

### Heat stress and pair-feeding alter ileal gene expression

Expression changes of key metabolic, integrity, and functional genes due to HS and PFTN are reported in Table 4. Ileal HSP 27 gene expression was increased 3-fold (*P* = 0.05) in HS pigs compared to both TN and PFTN pigs. A similar trend followed for HSP 70 (*P* < 0.01) and HSP 90AA (*P* = 0.15), although the magnitude of increase was different (15-fold for HSP 70 and 2.5-fold for HSP 90- α). Heat shock factor-1 (HSF1) gene expression was not different between treatments (*P* > 0.05). Hypoxia inducible factor 1- α (HIF1A) was also not different between treatments (*P* > 0.05); however there was a tendency for HIF 2 to be increased (*P* = 0.07) in HS pigs compared to both TN and PFTN pigs. Lactate degydrogenase A (LDHA) did not differ between treatments (*P* > 0.05); however pyruvate dehydrogenase kinase (PDK4) was increased 15% (*P* < 0.01) 15-fold in HS pigs compared to both TN counterparts. There were no differences between the three treatments (*P* > 0.05) in the gene abundances of sodium-glucose cotransporter-1 (SLC5A1), Na^+^/K^+^ ATPase (ATP1A1), AMP activated protein kinase- α (PRKAA2), glucose transporter 2 (SLCA2), citrate synthase (CS), glyceraldehyde phosphate dehydrogenase (GAPDH), hexokinase (HK1), or catalase (CAT).

**Table 4:** Effects of ad-libitum feed intake in thermal neutral conditions (TN; 21°C), ad-libitum feed intake in heat stress conditions (HS; 37°C), or pair-feeding in thermal neutral conditions (PFTN) on ileal mRNA abundance.

Gene ID		Treatment^1^			SE	*P-value*
		TN	HS	PFTN		
HSP27	Heat Shock Protein 27	0.50^x^	1.30^y^	0.50^x^	0.243	0.052
HSP70	Heat Shock Protein 70	0.33^a^	5.32^b^	0.33^a^	0.727	<0.01
HSP90AA	Heat Shock Protein 90-α	0.54	1.24	0.59	0.270	0.151
HSF1	Heat Shock Factor-1	5.36	7.78	5.24	0.161	0.447
HIF1A	Hypoxia Inducible Factor-a	1.79	2.55	1.99	0.424	0.419
HIF2	Hypoxia Inducible Factor-2	1.86^x^ ^y^	3.72^y^	1.41^x^	0.670	0.066
LDHA	Lactate Dehydrogenase A	2.83	2.31	1.44	0.630	0.296
PDK4	Pyruvate Dehydrogenase Kinase	0.53^a^	5.39b	0.41^a^	1.08	<0.01
SLC5A1	Sodium-glucose cotransporter-1	3.42	2.68	1.90	0.941	0.507
ATP1A1	Na^+^/K^+^ ATPase	3.38	4.40	1.94	0.879	0.155
PRKAA2	AMP Activated Protein Kinase-α	2.22	2.43	1.48	0.590	0.504
SLCA2	Glucose Transporter 2	2.54	2.50	1.24	0.732	0.376
CS	Citrate Synthase	0.08	0.03	0.08	0.026	0.553
HK1	Hexokinase-1	2.70	3.49	1.79	0.768	0.327
GAPDH	Glyceraldehyde phosphate dehydrogenase	2.53	1.56	1.89	0.466	0.315
CYCS	Cytochrome C oxidase	1.79	1.57	1.10	0.321	0.302
CAT	Catalase	1.49	2.07	1.62	0.512	0.695

^a,b^ *P* < 0.05,

^x,y^ *P* < 0.10,

n = 8/treatment

^1^All data normalized to the reference gene TOP2B

## Discussion

Heat stress and the reduced caloric intake associated with HS can alter intestinal integrity and function [11,12,20,31]. Herein, we utilized a short term HS exposure (12 hours, 37°C; 40% humidity) model in these growing pigs. We have previously reported that both HS and caloric restriction under TN conditions for 12 hours antagonize intestinal function and integrity [30]. Additionally, in these same pigs, the skeletal muscle proteome significantly changes in response to HS and pair-feeding [32]. However, what causes the functional changes in the gastrointestinal tract is poorly defined. Therefore, the present study utilized 2D-DIGE proteomics and targeted PCR to identify protein and mRNA changes that may help explain intestinal responses to HS. Ileum proteome analysis identified the relative abundance of 281 and 138 spots as different due to HS, compared to TN and PFTN treatments, respectively. However, only 20 proteins were different due to feed intake (thermal neutral verses pair-fed thermal neutral).

Herein, we have reported several heat shock proteins (HSP), including HSP 27, 65, 70, 90-α and 90-β that are upregulated due to HS compared to either TN or PFTN ileum samples. Gene abundance of HSPs 27, 70, and 90-α followed a similar pattern to the protein expression differences. Heat shock proteins are a diverse family of proteins that are important in the stress response as they act as chaperones and housekeepers and are involved in inhibition of apoptosis, regulation of cell development and differentiation, signal transduction as well as thermal tolerance, protection and recovery of misfolded, unfolded, or otherwise altered proteins due to elevated temperatures [33]. Previously, we have shown an increased protein and mRNA abundance of jejunum HSP27 [12] and HSP 70 in the intestine in both ileum and colon of HS pigs [11,20]. Further, stress-induced-phosphoprotein 1 was also upregulated due to HS in the present study. This protein is also known as Hsp70/Hsp90-organizing protein (HOP) which functions as a co-chaperone to link HSP 70 and HSP 90 together [34].

Another pathway that appeared to be induced by HS is the oxidative stress or antioxidant system pathway. Antioxidant status during disease is extremely important for protection of cells as well as defense against invading pathogens [35]. The down-regulation of Peroxiredoxin-1 during HS may suggest a decreased ability of animals to handle oxidative stress and reactive oxygen species. These results are similar to what Cruzen et al., reported in semitendinosus skeletal muscle of HS treated pigs [32]. Peroxiredoxins are antioxidant enzymes, which control and reduce cytokine-induced hydrogen peroxide, lipid hydroperoxides and peroxynitrites [36].

The intestine is generally physiologically hypoxic under normal conditions; however, several conditions can also induce hypoxia such as altitude sickness, ischemia/reperfusion injury, heat stress, as well as others [37]. Hypoxia-inducible factors (HIFs) are transcriptional regulators that play a role in oxygen homeostasis. There are three subunits involved in oxygen regulation including HIF 1-α, HIF 2-α, and HIF 3-α that require hydroxylation by prolyl-4-hydroxylases to be targeted for ubiquitination and subsequent proteolysis to remain inactive [38]. There are three prominent prolyl-4-hydroxylase domains (PHD1, PHD2, and PHD3) with the PHD2 involved in HIF 1-α regulation [39]. In previous studies by our group, we have shown an upregulation of HIF 1-α in the intestine under HS conditions [11]. Our current data suggest that lesser abundance of prolyl-4-hydroxylase beta in HS pig ileum samples that may indicate a protective effect for the intestine, and may also help explain an up-regulation in HIF, as it would be in a more active state. Hypoxia-inducible factor prolyl-4-hydroxylase inhibitors have been shown to prevent oxidative death and ischemic injury [40]. This is in partial agreement with the measured gene expression of both HIF genes being higher in HS pigs.

Although intestinal glucose transport and energetic genes did not differ due to HS, multiple metabolic proteins were downregulated due to heat stress, independent of feed intake. Isocitrate dehydrogenase (IDH) is a Krebs cycle enzyme responsible for converting isocitrate to isoglutarate while also producing NADPH. This enzyme was dramatically decreased in HS pigs. Aside from its role in the TCA cycle, IDH also has other functions and its product, 2-oxoglutarate, is required for HIF 1-α hydroxylation and activation [41]. Heat stress also down regulated three other glycolytic/gluconeogenic pathway enzymes, which included fructose bisphosphate aldolase, alpha-enolase, and glyceraldehyde-3-phosphate dehydrogenase (GAPDH). These enzymes are oxioreductases mainly known for their involvement in glycolysis. Recent research suggests that there are several non-glycolytic functions of GAPDH such as regulating oxidative stress and cellular redox status [42].

We have previously demonstrated increases in blood glucose, glucose transport mechanisms [11] and decreases in brush border enzyme activities in pigs heat-stressed for 24 h [43]. Therefore, metabolically, both HS and presumably its associated endotoxin-induced inflammation have been shown to shift post-absorptive fuel selection from oxidative phosphorylation to glycolytic metabolism. Interestingly HIF 1-α transcribes genes involved in glycolytic metabolism [44], including the three proteins identified here. To further the idea that heat-stressed mammals conserve glucose, we observed an increase in gene expression of pyruvate dehydrogenase kinase (PDK4). This enzyme inhibits the pyruvate dehydrogenase complex and conserves glucose by reducing its conversion to pyruvate and vious HS studies which observe increases in PDK4 in muscle [45].

Intriguingly, we also saw a reduction in peptidyl-prolyl isomerase A (Pin1 or Cyclophilin A) in two different protein spots. Pin1 is one of the main cyclophilins in mammalian cells and possesses peptidyl-prolyl isomerase activity. Peptidyl-prolyl isomerase catalyzes the isomerization of peptidyl-prolyl peptide bonds. Pin1 is also important for phosphorylation-dependent signaling pathways including apoptosis, and the cell cycle. Under normal conditions, Pin1 is abundant in enterocytes located in the villi of the small intestine, while under disease conditions it is only found near the crypt regions [46]. Several studies have shown that expression of Pin1 is controlled by HIF 1-α and that upregulation of the gene induces activation of ERK signaling, thus increasing cell proliferation [47].

Heat Stress reduces internal integrity and increases macromolecule flux across the intestinal epithelium [11,20]. Although no tight junction protein spots were picked and identified as differently different, multiple proteins involved in cell structure, integrity and signal transduction were also altered due to HS. Vimentin is a type III intermediate filament protein normally expressed in mesenchymal cells and in the intestines it is involved in cell migration. It, along with other microfilaments comprises the cytoskeleton. Depending on the species, vimentin is mainly expressed in the lamina propria of the intestinal mucosa as well as at the base of crypts [48]. However, it is not significantly expressed in epithelial cells themselves. Manipulation of the cell plasma membrane and its associated proteins is necessary for pathogen survival, and research has shown vimentin to be a binding target for pathogens such as porcine reproductive respiratory syndrome virus [49]. Thus, the upregulation of vimentin may be associated with changes in intestinal integrity as vimentin gene expression has been shown to increase during bacterial infection [50]. Cofilin was decreased in the HS ileum samples compared to both TN and PFTN treatments. Decreased intestinal integrity has been correlated with cofilin dephosphorylation, possibly implicating it in disruption of tight junctions in Caco-2 cells [51]. When cofilin is dephosphorylated, this causes F-actin to rearrange and appears to be independent of MLCK activation [51]. Another cytoskeleton element that is changing due to HS is calponin. Compared to animals on a similar plane of nutrition, HS caused a decrease in calponin-1 abundance. Calponin is a calcium-binding inhibitor of the ATPase activity of myosin, decreasing the ability of smooth muscle to contract. Therefore, a decrease in calponin may indicate an increase in smooth muscle contraction, as myosin ATPase activity would be less inhibited.

Rho GDP-dissociation inhibitor 1 or α is a member of the Rho GDP-dissociation inhibitor (RhoGDI) family proteins, which negatively regulate Rho-family GTPases and control Rho protein homeostasis. RhoGDIs inhibit GTPase functions including cytokinesis, phagocytosis, actin dynamics, and cell motility. In cell culture models of cancer, loss of RhoGDI increases apoptosis in a caspase-dependent manner [52]. Effects of RhoGDI’s on epithelial cells are largely unclear, but may be implicated in reducing epithelial cell integrity and increased permeability during chronic inflammation as seen during inflammatory bowel disease in humans [53].

Part of the objective of the study was to define the direct and indirect effects of feed intake. Only three proteins identified, were altered due to a short-term reduction in feed intake. Prolyl-hydroxylase was decreased due to feed intake, while vimentin and HSP 70 were increased. These data suggest that PFTN pigs were experiencing some level of hypoxia and stress [54]. Food deprivation and decreased feed intake has been shown to negatively affect intestinal function under various disease and starvation states [55,56]. A previous proteomic study in mice where animals were starved for 12 h showed that after a short period, enzymes involved in glycolysis and energy metabolism were decreased [57]. Although the timing was similar to the current study, our PFTN pigs did have access to a limited amount of feed twice a day.

## Conclusion

In conclusion, these data illustrate an increase in the heat shock response and hypoxia in the small intestine of pigs subjected to 12 h of a severe heat-load. Even with an increase in intestinal stress, these animals appear to have a reduced ability to combat ROS. With that, several metabolic enzymes are also reduced, many of which are involved in the glycolytic or TCA pathways indicating a change in metabolic priorities. This may be due to a change in substrate priority, or a shift in pathway intermediates to utilize for dealing with an increased ROS load. Immune function is also altered with the immunoproteasome most affected by HS. Lastly, cell structure and signal transduction were negatively affected by HS and several proteins, especially those related to intermediate filaments (Table 5).

**Table 5:** Categorization and general function information for proteins identified as differentially expressed.

**Metabolism**	**General Function**
Isocitrate Dehydrogenase [NADP], mitochondrial	Converts isocitrate to oxoglutarate (Krebs cycle)
Chain A, Fructose 1,6-Bisphosphate Aldolase	Splits F 1,6 BP to DHAP and GAP, glycolysis
Glyceraldehyde-3-phosphate dehydrogenase (phoshorylating)	Converts GADP to 1,3 BPG in Glycolysis
Alpha-enolase	Glycolysis, hypoxia tolerance, growth
**Stress Response**	**General Function**
Endoplasmin precursor (Heat shock protein 90 kDa β-member 1)	Chaperone, protein folding, ATPase
Heat shock protein β-1 (Heat Shock Protein 27)	Stress resistance, actin organization, inducible
Heat shock protein HSP 90-α	Molecular chaperone, ATPase
Stress-induced-phosphoprotein 1 (Hsc70/Hsp90-organizing protein)	Mediates association of molecular chaperones HSC70 and HSP90
Heat shock 70kDa protein 8 (2 spots)	Chaperone, protein folding, ATPase
Heat shock protein 65	Immunodominant antigen, immune response
**Oxidative Stress/Immune System**	**General Function**
Peroxiredoxin-1	Antioxidant, reduces ROS
Peptidyl-prolyl cis-trans isomerase A (Cyclophilin A) (2 spots)	Accelerates protein folding, immunosuppression
Prolyl 4-hydroxylase beta polypeptide	Prolyl hydroxylation of HIF and preprocollagen
Proteasome activator PA28 alpha subunit (PSME1)	Proteasome, immunoproteasome
Proteasome activator complex subunit 2	Immunoproteasome assembly, antigen processing
Immunoglobulin γ-chain	Antibody
**Cell Structure/Signal Transduction**	**General Function**
Vimentin-like	Intermediate filament, cell structure
Rho GDP-dissociation inhibitor 1 (GDIR-1)	Rho protein homeostasis, cell motility
Neurofibromin (NF-1)	Negative regulator of Ras pathway
Cofilin-1	Actin-modulating protein, depolymerizes actin

## Supporting Information

S1 Fig 2D-Western blot protein identification confirmations. All spots identified using 2D-Western blots were in the same molecular weight and pH range as the protein spot in the 2D-DIGE gel. A secondary control was performed for each secondary used. Antibodies and concentrations are listed in S1 Table.
(DOCX)

S1 Table Primary and secondary antibody dilutions and manufacture information of the antibodies used for confirmation of select proteins identified in the 2D Difference In Gel Electrophoresis analysis. (DOCX)

S2 Table Primer Sequences. (DOCX)

S3 Table Protein identified with individual peptides and Mowse Score. (DOCX)
