# Supplementary material for: Acute Heat Stress and Reduced Nutrient Intake Alter Intestinal Proteomic Profile and Gene Expression in Pigs
Source: PLoS One. 2015 Nov 17;10(11):e0143099. doi: 10.1371/journal.pone.0143099 (PMC4648527; doi:10.1371/journal.pone.0143099)
Supplement: S1 Fig — All spots identified using 2D-Western blots were in the same molecular weight and pH range as the protein spot in the 2D-DIGE gel. A secondary control was performed for each secondary used. Antibodies and concentrations are listed in S1 Table. (DOCX) [file pone.0143099.s001.docx]

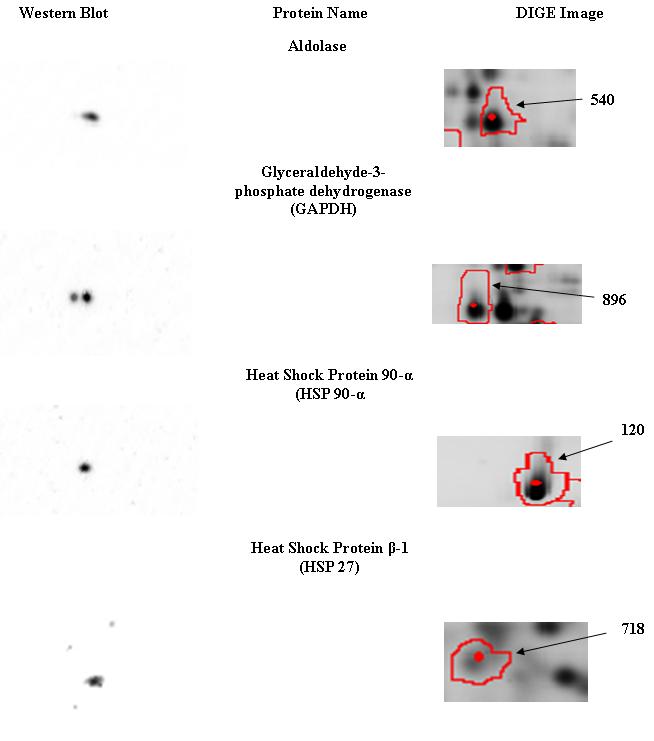


**S1 Fig.** 2D-Western blot protein identification confirmations. All spots identified using 2D-Western blots were in the same molecular weight and pH range as the protein spot in the 2D-DIGE gel. A secondary control was performed for each secondary used. Antibodies and concentrations are listed in Table S1.
