# Supplementary material for: Acute Heat Stress and Reduced Nutrient Intake Alter Intestinal Proteomic Profile and Gene Expression in Pigs
Source: PLoS One. 2015 Nov 17;10(11):e0143099. doi: 10.1371/journal.pone.0143099 (PMC4648527; doi:10.1371/journal.pone.0143099)
Supplement: S1 Table — (DOCX) [file pone.0143099.s002.docx]

**S1 Table:** Primary and secondary antibody dilutions and manufacture information of the antibodies used for confirmation of select proteins identified in the 2D Difference In Gel Electrophoresis analysis.

| **Protein** | **Primary Antibody**  **Dilution**  **Type**  **Manufacture** | **Secondary Antibody**  **Dilution**  **Manufacture** |
| --- | --- | --- |
| Glyceraldehyde-3-phosphate dehydrogenase (GAPDH) | Anti-GAPDH  1:1,000  Mouse Monoclonal  Biochain Y3322GAPDH | Anti-mouse IgG  1:10,000 NB7544  Novus Biologicals |
| Aldolase | Anti-Aldolase  1:20,000  Rabbit monoclonal  Abcam AB169544 | Anti-rabbit IgG  1:10,000  81-6180  Zymed Laboratories |
| Heat Shock Protein 70 (HSP 70) | Anti-HSP70  1:1,000  Mouse Monoclonal  Novus NB110-96427 | Anti-mouse IgG  1:10,000  7076S  Cell Signaling |
| Heat Shock Protein 27 (HSP 27) | Anti-HSP27  1:1,000  Rabbit Monoclonal  Abcam AB109376 | Anti-rabbit IgG  1:10,000  7074S  Cell Signaling |
| Heat Shock Protein 90-α (HSP 90-α) | Anti-HSP90A  1:1,000  Rabbit Monoclonal  Cell Signaling 8165S | Anti-rabbit IgG  1:10,000  7074S  Cell Signaling |
