# Supplementary material for: Acute Heat Stress and Reduced Nutrient Intake Alter Intestinal Proteomic Profile and Gene Expression in Pigs
Source: PLoS One. 2015 Nov 17;10(11):e0143099. doi: 10.1371/journal.pone.0143099 (PMC4648527; doi:10.1371/journal.pone.0143099)
Supplement: S2 Table — (DOCX) [file pone.0143099.s003.docx]

**S2 Table:** Primer Sequences

| **Gene Name** | **Sense (5’- 3’) - Forward** | **Antisense (5’- 3’) - Reverse** |
| --- | --- | --- |
| Heat Shock Protein 27 (HSP27) | AGCTGACGGTCAAGACCAA | AAATGAAGCCGTGCTCATCC |
| Heat Shock Protein 70 (HSP70) | CCTGGAGGCCTATGTTTTCCA | GCACCTGTCCTCTTCAGGAA |
| Heat Shock Protein 90- α (HSP90AA1) | TCTGCTGTCTTCTGGCTTCA | GTGGGGTCGTCCTCATCAA |
| Heat Shock Factor-1 (HSF1) | GCAGCTCCTGGAGAACATCA | ACACTGTCCTGGCGAATCTTTA |
| Hypoxia Inducible Factor- α (HIF1A) | GAACAGAATGGAACGGAGCAA | TGATTGCCCCAGGAGTCTAC |
| Hypoxia Inducible Factor 2 (HIF2) | AGCTCTTCGCCATGGATACA | TCCAGGTCCAGCTCATTGAA |
| Lactate Dehydrogenase A (LDHA) | CAACATGGCAGCCTTTTCCTTA | CCAGCCTAGAGTTTGCAGTCA |
| Pyruvate Dehydrogenase Kinase (PDK4) | TTGGCAGCATTGACCCAAAC | CTGATTACAGAGCATCTTGGAACAC |
| Sodium-glucose cotransporter-1 (SLC5A1) | GGCTGTTCCAACATTGCCTA | CAACATGACCGACAGCATCA |
| Na^+^/K^+^ ATPase (ATP1A1) | GCTGACACGACGGAAAATCA | CTGCAATTCTGGACAGAGCAA |
| AMP Activated Protein Kinase- α (PRKAA2) | GAACATCAACTGACAGGCCATA | CTAAACTGCGAATCTTCTGCCTA |
| Glucose Transporter 2 (SLCA2) | TCATCAGCTGGCCATTGTCA | GCTCATGATTGCCCAGGAGAA |
| Citrate Synthase (CS) | ACCATGAAGGTGGCAGTCTA | GCTGCTGCAAAGGACAAGTA |
| Hexokinase (HK1) | CTGGCCTATTACTTCACCGAAC | AGCCGCATGGCATAGAGATA |
| Glyceraldehyde-3-phosphate dehydrogenase (GAPDH) | GGGCTGCCCAGAACATCA | CCGTTGAGCTCAGGGATGAC |
| Catalase (CAT) | GCTTCAACAGTGCCAACGAA | CGTTTCCTCTCCTCCTCATTCA |
| Topoisomerase (DNA) II beta (TOP2B) | CGGCAGGAGAACATCCAAAA | GGGAAGAGGTCCACATCTGAA |
