# Supplementary material for: Acute Heat Stress and Reduced Nutrient Intake Alter Intestinal Proteomic Profile and Gene Expression in Pigs
Source: PLoS One. 2015 Nov 17;10(11):e0143099. doi: 10.1371/journal.pone.0143099 (PMC4648527; doi:10.1371/journal.pone.0143099)
Supplement: S3 Table — (DOCX) [file pone.0143099.s004.docx]

**S3 Table:** Protein identified with individual peptides and Mowse Score

| Spot ID | Protein ID | Species | Accession | pI | Mass (kDa) | Coverage  % | Matched Peptides (all +1 charge) | Mowse  Score |
| --- | --- | --- | --- | --- | --- | --- | --- | --- |
| 364 | Prolyl 4-hydroxylase beta polypeptide | *Sus scrofa* | AET99218.1 | 4.78 | 56.8 | 9% | VDATEESDLAQQYGVR  YKPESAELTAEAIR  NFEEVAFDAR  TVIDYNGER | 226 |
| 515 | Isocitrate dehydrogenase [NADP], mitochondrial | *Sus scrofa* | P33198.1 | 8.74 | 47.9 | 12% | YFDLGLPNR  NILGGTVFR  HAHGDQYK  IWYEHR  SSGGFVWACK  GKLDGNQDLIR | 153 |
| 334 | Vimentin-like | *Sus scrofa* | XP_005668163.1 | 5.06 | 53.7 | 8% | SYLTSSPGGVYATR  FADLSEAANR  ISLPLPNFSSLNLR | 124 |
| 158 | Heat Shock Protein 90-alpha | *Sus scrofa* | NP_999138.1 | 4.93 | 85.1 | 7% | HFSVEGQLEFR  RAPFDLFENR  GVVDSEDLPLNISR  LGIHEDSQNR  DQVANSAFVER | 288 |
| 823 | Peptidyl-prolyl cis-trans isomerase A-like | *Otolemur garnettii* | XP_003797634.1 | 8.33 | 18.1 | 14% | IIPGFMCQGGDFTR  FEDENFVLK | 82 |
| 825 | Peptidyl-prolyl cis-trans isomerase A | *Sus scrofa* | NP_999518.1 | 8.34 | 18.1 | 19% | VNPTVFFDIAVDGEPLGR  IIPGFMCQGGDFTR | 87 |
| 718 | Heat Shock Protein beta-1 | *Sus scrofa* | NP_001007519.1 | 6.23 | 23.0 | 24% | SPSWDOFRDWYPAHSRLFDQAFGLPR  QLSSGVSEIQQTADR  QDEHGFISR | 215 |
| 896 | Glyceraldehyde-3-phosphate dehydrogenase (phosphorylating) | *Sus scrofa* | DEPGG3 | 6.90 | 35.9 | 12% | AITIFQERDPANIK  VPTPNVSVVDLTCR  LISWYDNEFGYSNR | 191 |
| 120 | Endoplasmin Precursor | *Mus musculus* | NP_035761.1 | 4.74 | 92.7 | 9% | FAFQAEVNR  NKEIFLR  SILFVPTSAPRGLFDEYGSK  GVVDSDDLPLNVSR  LGVIEDHSNR  IYFMAGSSR  EAESSPFVER | 311 |
| 739 | Rho GDP-dissociation inhibitor 1 | *Bos tarus* | NP_788823.1 | 5.12 | 23.5 | 11% | VAVSADPNVPNVVVTR  YIQHTYR | 55 |
| 192 | Neurofibromin-1 | *Rattus norvegicus* | NP_036741.1 | 6.94 | 32.0 | 0% | NMFGETLHK  TCAPGASLR | 38 |
| 235 | Heat shock Protein 70 | *Sus scrofa* | XP_004459596.1 | 5.37 | 71.0 | 5% | TTPSYVAFTDTER  TVTNAVVTVPAYFNDSQR  DAGTIAGLNVLR  ARFEELNADLFR | 339 |
| 238 | Heat shock Protein 70 | *Mus musculus* | NP_998931.1 | 5.92 | 71.3 | 10% | TTPSYVAFTDTER  TVTNAVVTVPAYFNDSQR  DAGTIAGLNVLR  ARFEELNADLFR | 237 |
| 333 | Heat Shock Protein 65 | *Mus musculus* | CAA38762.1 | 5.91 | 61.1 | 6% | ISSVQSIVPALEIANAHR  AAVEEGIVLGGGCALLR | 95 |
| 440 | Alpha Enolase | *Sus scrofa* | P19140.2 | 6.37 | 47.6 | 15% | AAVPSGAS TGIYEALELR  IGAEVYH NLK  VVIGMDVAASEFYR  YISPDQLADLYK  LAQSNGWGVMVSHR | 324 |
| 540 | Chain A, Fructose 1,6-Bisphosphate Aldolase | *Oryctolagus cuniculus* | 1ADO_A | 8.30 | 39.6 | 12% | ELSDIAHR  ADDGRPFPQVIK  YSHEEIAMATVTALR  AAQEEYVKR | 173 |
| 293 | Stress-induced Phosphoprotein 1 | *Bos tarus* | NP_001030569.1 | 6.08 | 63.1 | 3% | LDPQNHVLYSNR  ELIEQLR | 69 |
| 812 | Cofilin-1 | *Sus scrofa* | NP_001004043.1 | 8.16 | 18.8 | 13% | MLPDKDCR  HELQANCYEEVKDR | 103 |
| 620 | Calponin-1 | *Sus scrofa* | NP_999043.1 | 8.91 | 33.4 | 10% | YDHQQEQELR  FASQQGMTAYGTR  GMTVYGLPR | 128 |
| 693 | Proteasome activator PA28 alpha subunit | *Sus scrofa* | AAQ04767.1 | 5.45 | 24.2 | 14% | VDVFREDLCTK  QPHVGDYRQLVHELDEAEYR | 119 |
| 677 | Proteasome activator complex subunit 2 | *Sus scrofa* | NP_999444.1 | 5.41 | 27.4 | 9% | ETHVMDYRALVHERDEAAHGELR | 148 |
| 342 | Immunoglobulin gamma-chain | *Sus scrofa* | AAA51295.1 | 6.71 | 51.9 | 6% | LVESGGGLVQPGGSRL  TAPSVYPLAPCGR | 132 |
| 711 | Peroxiredoxin-1 | *Rattus norvegicus* | NP_476455.1 | 8.27 | 22.0 | 5% | QITINDLPVGR   LSSGLPEAR   EDLGNPSADLK   YIYEIAR | 45 |
